# Adiabatic superconducting cells for ultra-low-power artificial neural networks

**DOI:** 10.3762/bjnano.7.130

**Published:** 2016-10-05

**Authors:** Andrey E Schegolev, Nikolay V Klenov, Igor I Soloviev, Maxim V Tereshonok

**Affiliations:** 1 Lomonosov Moscow State University, Physics Faculty, Moscow, 119991, Russia; 2 Moscow Technical University of Communications and Informatics (MTUCI), Moscow, 111024, Russia; 3 Lomonosov Moscow State University Skobeltsyn Institute of Nuclear Physics, Moscow, 119991, Russia; 4 N.L. Dukhov All-Russia Research Institute of Automatics, 127055, Moscow, Russia; 5 Moscow Institute of Physics and Technology, State University, Dolgoprudniy, Moscow region, Russia; 6 Lukin Scientific Research Institute of Physical Problems, Zelenograd, Moscow, 124460, Russia

**Keywords:** adiabatic superconductor cells, artificial neural networks, energy efficiency, Josephson effect, superconductivity

## Abstract

We propose the concept of using superconducting quantum interferometers for the implementation of neural network algorithms with extremely low power dissipation. These adiabatic elements are Josephson cells with sigmoid- and Gaussian-like activation functions. We optimize their parameters for application in three-layer perceptron and radial basis function networks.

## Findings

Artificial neural networks (ANNs) are famous for their application in the fields of artificial intelligence and machine learning [1]. The future of cellular and satellite communications, radar systems, deep sea and space exploration will likely be closely related to the capability of ANNs to provide effective solutions to problems such as classification and recognition of signals or images [2–6]. The important features of receiving systems exploited in such areas are high energy efficiency, sensitivity and variability in signal processing. This makes the utilization of superconducting electronic constituents a natural choice.

Superconducting digital receiving and computing are emerging technologies in high-speed/high-frequency electronic applications markets [7]. The advantages of a superconducting digital RF receiver [8] are high sampling rate and quantum precision of quantization, allowing direct digitization of incoming wideband RF signals without conventional channelization and downconversion. The combination of such receivers with highly sensitive, tunable, active, superconducting antennas [8–11] and ANNs provides an opportunity for the development of a cognitive radio correlation receiver. Unfortunately, among superconducting ANNs [12–13], those for signal classification and recognition are less developed.

A solution for the recognition problem by employing perceptron ANNs was sought in earlier works with SQUID-based neuron switching [14–15] in the resistive state. In subsequent variations [16–17], this feature was found to drastically reduce the energy efficiency of the superconducting circuit. In another recent approach to multilayer perceptron, SQUIDs were utilized as nonlinear magnetic flux transducers, allowing the ANN to persist in the superconducting state [18]. The implemented neuron scheme is quite analogous to the quantum flux parametron (QFP) [19–20] – the basic cell of a superconducting logic circuit, known for their high energy efficiency. It was experimentally shown that QFP-based circuits operated in the adiabatic regime can outperform their semiconductor counterparts with respect to energy efficiency by seven orders of magnitude (including the power required for superconducting circuit cooling) [21–24]. While the activation function of the QFP neuron was not analyzed in [18], our assay shows that it is not well suited for the chosen type of network.

The activation function commonly has a highly nonlinear form and is a key characteristic of a neuron. Note that semiconductor-based neurons contain at least approximately 20 transistors due to the lack of nonlinearity between the transistor current and voltage. The typical implementation of an ANN is based on field-programmable gate arrays (FPGAs), making them relatively slow and hardware/power consumable. The basic element of a superconducting circuit is the nonlinear Josephson junction, which is about three orders faster than a conventional transistor. In contrast to semiconductor neurons, the superconducting one typically consists of just a few (two or three) Josephson junctions. This presents a distinct opportunity for the development of energy efficient, high density, fast superconducting ANNs for cognitive receiving systems.

It was shown that a Josephson structure (e.g., a bi-SQUID or a SQIF) transfer function can be precisely designed by combining basic SQUID cells with known characteristics [25–28]. In this letter we describe designs for superconducting neurons with sigmoid- and Gaussian-like shapes for the activation functions inspired by these works. Being based on a simple parametric quantron cell, our neurons allow an ANN to be operated in an extremely energy efficient, adiabatic regime. The neurons are proposed for perceptron and radial basic function (RBF) ANNs, which solve the signal recognition and identification problems, respectively. The complexity of these networks could be increased with further development of nanotechnology [29] with the implementation of nanoscale Josephson junctions (e.g., on the basis of variable thickness bridges [30]). Finally, comparison of the probability of error curves for RBF ANNs based on the proposed neuron with those based on an ideal neuron with a Gaussian activation function is presented.

## Sigma-cell: the basic element for a multilayer perceptron

A multilayer perceptron (MLP) is a feed-forward ANN model that maps input data onto a set of outputs [1]. An MLP consists of multiple layers of nodes in a directed graph with each layer fully connected to the next one. Each node is treated as a neuron whose activation function usually has a sigmoid-like shape.

**Figure 1:**
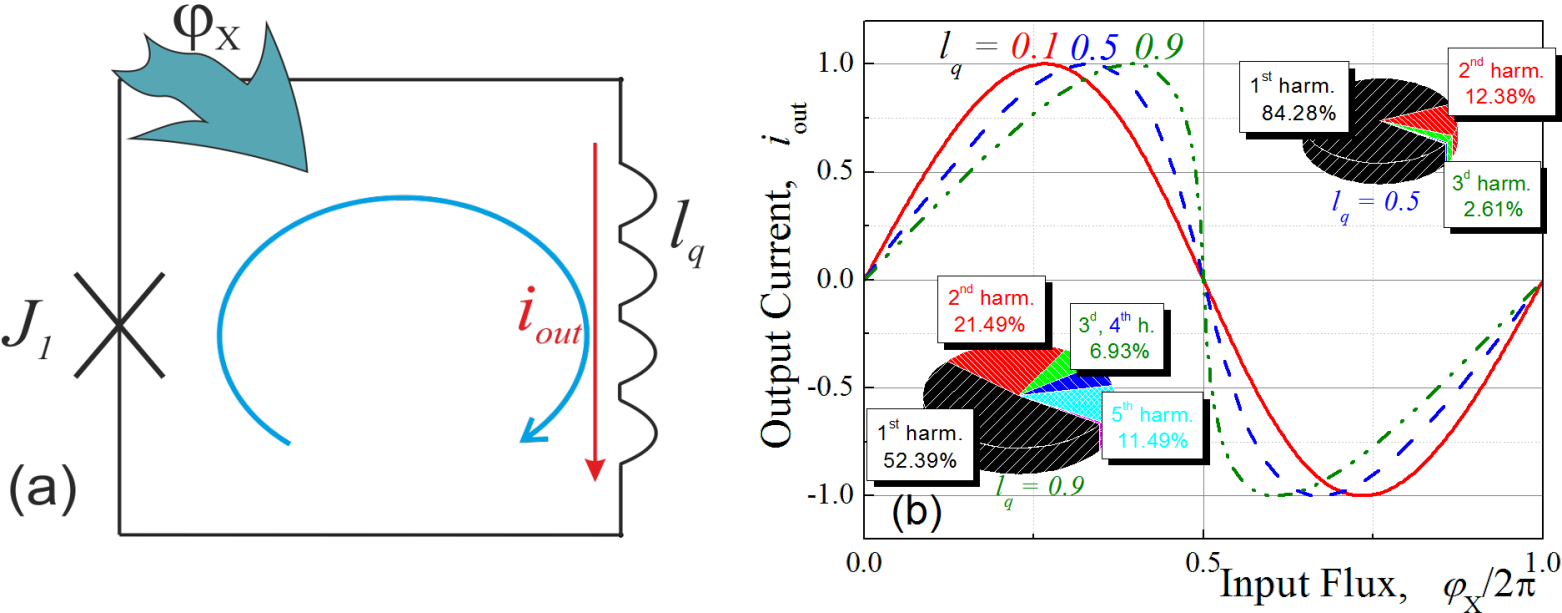
(a) Principle scheme for a potential quantron. (b) Quantron flux-to-current transfer function for different values of the normalized ring inductance *l*_q_; insets show the harmonic amplitudes for selected curves.

We start our pursuit of the MLP artificial neuron with an analysis of a simple quantron (or single-junction superconducting interferometer, Figure 1a) transfer function. This function links the applied magnetic flux, Φ_X_, with current flowing (*I*_out_) in a superconducting loop of inductance, *L*_q_. Hereafter, we use the normalization of inductance, *l*_q_ = 2π*I*_c_*L*_q_/Φ_0_ (where Φ_0_ is the magnetic flux quantum, *I*_c_ is the junction’s critical current), magnetic flux, φ_X_ = 2πΦ_X_/Φ_0_, and current, *i*_out_ = *I*_out_ /*I*_c_.

The phase balance for the quantron loop and the relationship between the current through the Josephson junction and its phase, φ, are as follows:

[1]
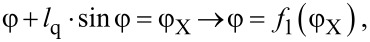


[2]
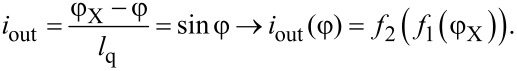


One can represent output current as a parametric function [25] and then plot the nonlinear flux-to-current characteristic, as shown in Figure 1b. Note that the resulting transfer function is non-sinusoidal and amplitudes of its higher harmonics increase with increasing inductance, *l*_q_.

**Figure 2:**
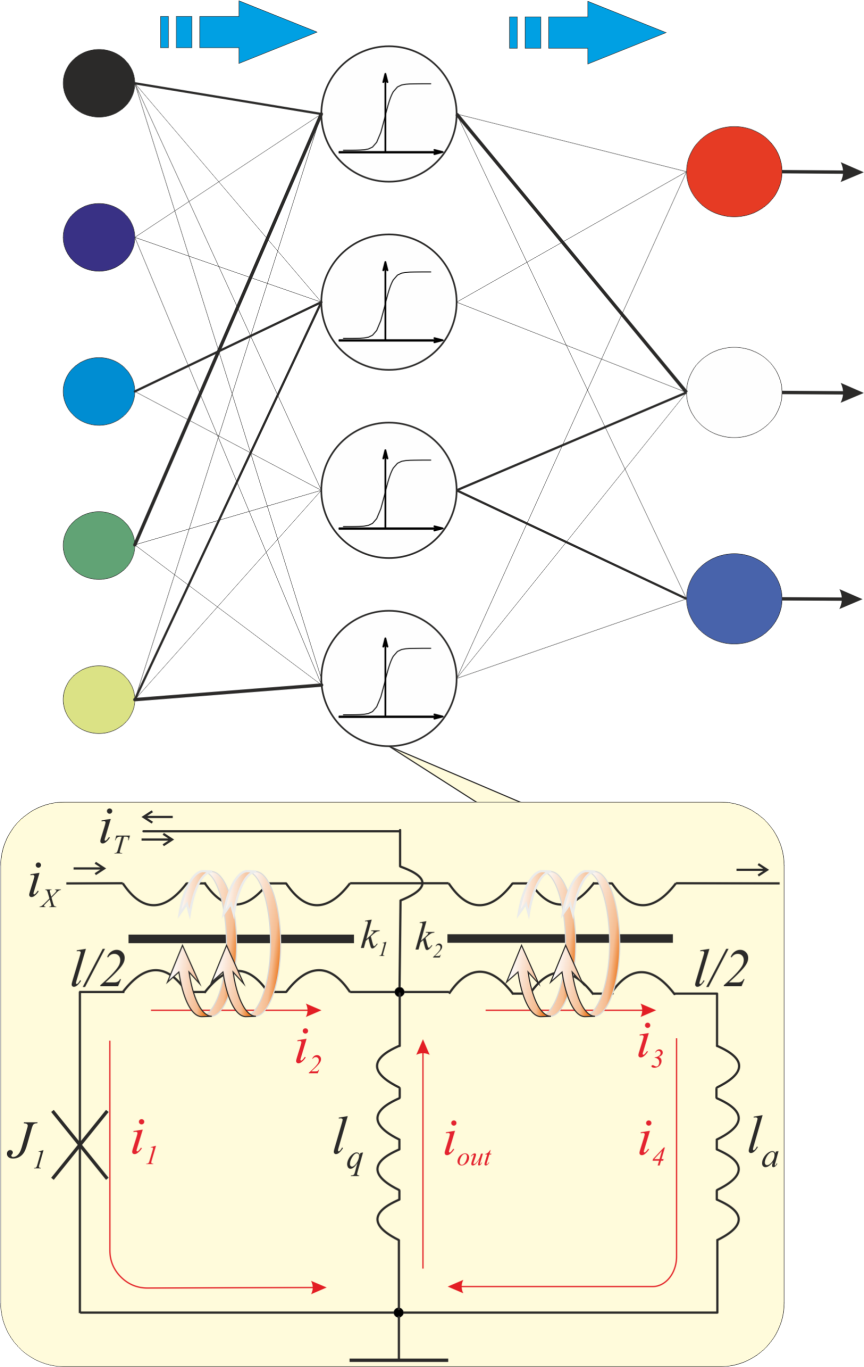
Principle scheme of a three-layer perceptron conceived as layers of connected nodes (with different weights in the general case) in a directed graph, and the suggested sigma cell with sigmoid-like flux-to-current transformation on the basis of a quantron and superconducting ring. Intersecting connections can be realized in the "magnetic domain" via an inductance *l*_q_ using a technique described in [18].

A sigmoid function is most suitable mathematically for the solution of the image or pattern recognition problems by means of MLP. One can provide this form of a flux-to-current transformation by combining the transfer function of the quantron with a linear dependence, which is provided by a simple superconducting ring. The principal scheme of the resulting sigma cell (or s-cell) as a part of a three-layer perceptron is presented in Figure 2. The magnetic flux is induced by the excitation current *I*_X_ in the control line, which is magnetically coupled to the quantron and the linear cell through mutual inductances *k*_1_ and *k*_2_, respectively. We shall assume for simplicity that the quantron contains inductances *l*_q_ and *l*/2, the superconducting ring – *l*_q_, *l*/2, *l*_a_. The current *i*_T_ (see Figure 2) allows the operating point to be set.

For analysis of the proposed cell flux-to-current transformation, one can write equations similar to Equation 1 and Equation 2. Here the phase balance and Kirchhoff's rule for the circuit considered give us the following expressions:

[3]
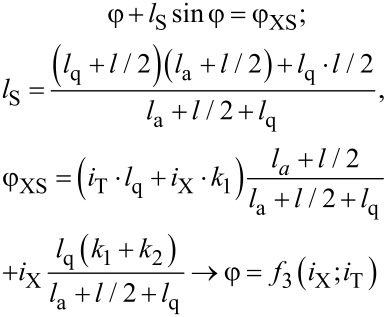


[4]
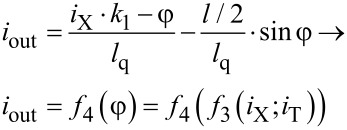


**Figure 3:**
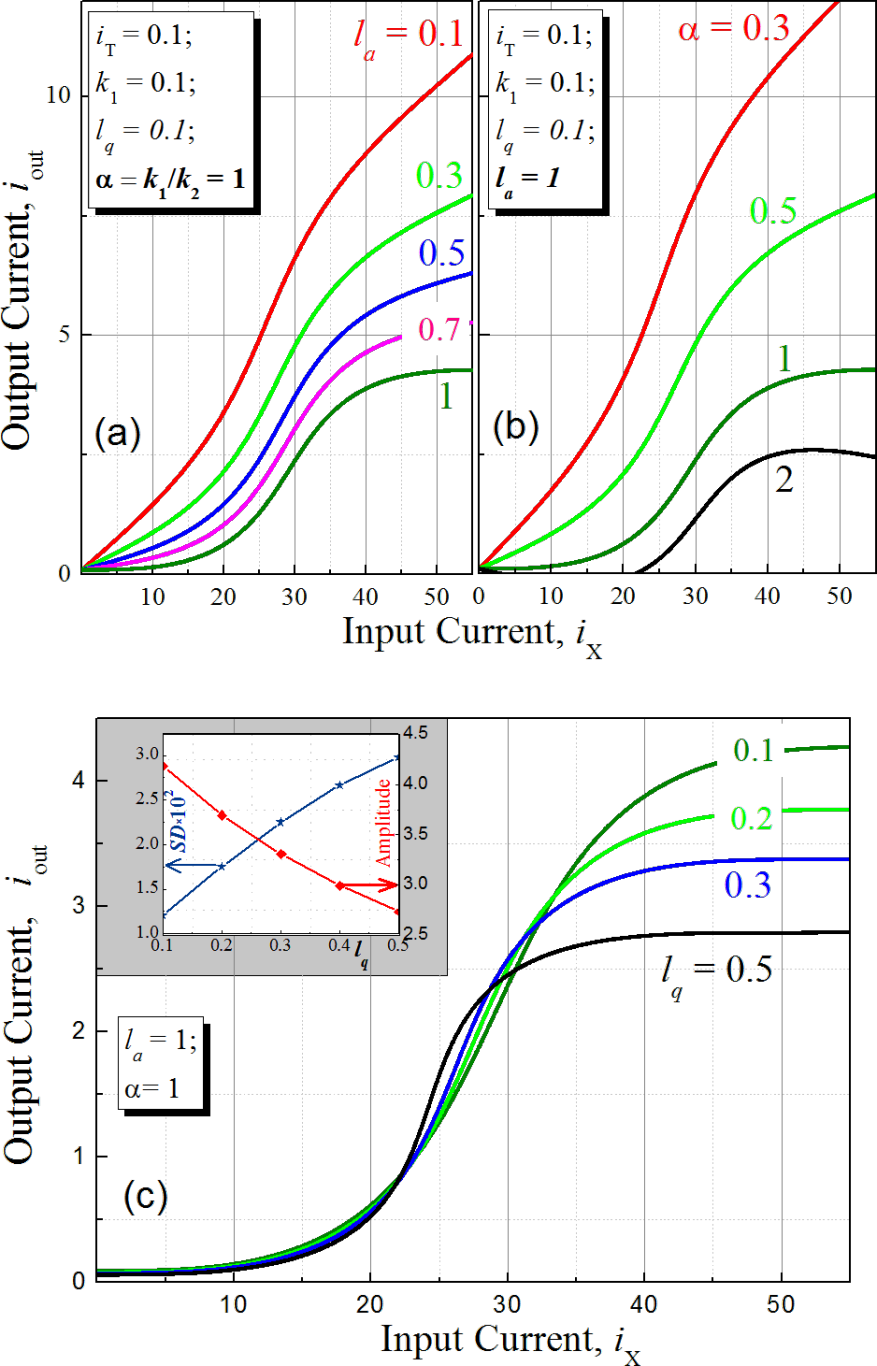
(a), (b) Flux-to-current characteristics of the sigma cell for different parameters of the superconducting ring *l*_a_ and α = *k*_1_/*k*_2_ at *l* = 0.6. (c) Sigma cell flux-to-current transfer function for a set of values of the inductance *l*_q_. The inset shows the amplitude of the transfer function and its standard deviation from the sigmoid function (×100).

Note that the expression for the output signal (Equation 4) contains a term with linear dependence on the input current *i*_X_. The resulting sigma cell flux-to-current transfer function is presented in Figure 3. An increase in the normalized inductance of the superconducting ring (at fixed quantron parameters) reduces the slope of the overall characteristic. The same effect can be obtained by decreasing the coupling of this ring with the control line. The figure shows that the overall slope practically disappears at *l*_a_ = 1, *l* = 0.6 and *k*_1_ = *k*_2_ = 0.1, which therefore is a preferred set of parameters for the physical implementation of the MLP neuron. Consistency of the obtained flux-to-current transformation with the sigmoid function can be tuned further by variation of the quantron inductance *l*_q_.

## Gauss cell: the basic element for a probabilistic network

The identification of different sources is a difficult problem in cognitive signal processing. MLP is the most frequently used for its solution. However, this type of neural network does not provide a probabilistic interpretation of the classification results and requires rather lengthy training [5–6]. An RBF-based network or a probabilistic network lack these disadvantages. Here, a decision requires the estimation of the probability density function for each class of radio signal sources, and so the basic cell has to provide a Gaussian-like transfer function.

**Figure 4:**
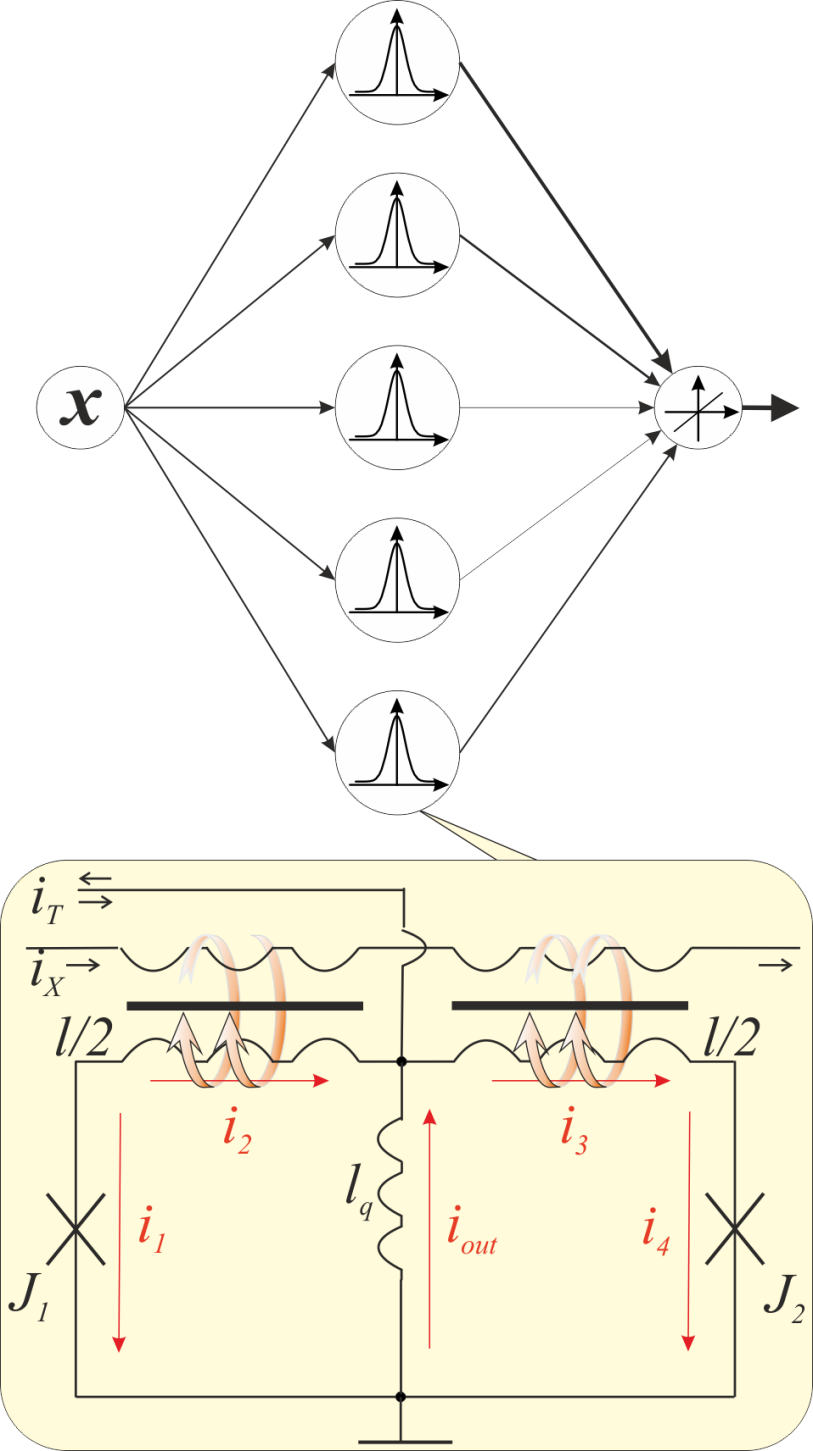
Principle scheme of an RBF neural network (where the output is a linear combination of radial basis functions of input *x* and neuron parameters) and suggested Gauss cell with Gaussian-like flux-to-current transformation.

The principal scheme of the proposed Gauss cell (or G-cell) is presented in Figure 4. Its design can be qualitatively understood as the connection of two s-cells in order to obtain a bell-shaped transfer function from two sigmoid functions. Note that the resulting scheme is quite analogous to the above mentioned QFP. The cell is a two-junction interferometer with a total normalized inductance *l,* composed of two Josephson junctions *J*_1_ and *J*_2_ shunted by inductance *l*_q_. Once again, the excitation current *I*_X_ is applied to a control line, which is magnetically coupled to the symmetrical arms of the interferometer.

One can write the equations for a Gauss cell by analogy with Equation 1 and Equation 2 in terms of the sum and difference phases, θ = (φ_2_ + φ_1_)/2; ψ = (φ_1_ − φ_2_)/2:

[5]
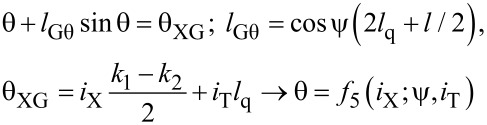


[6]
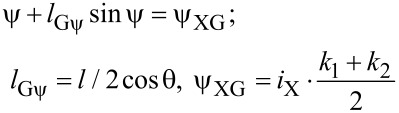


[7]
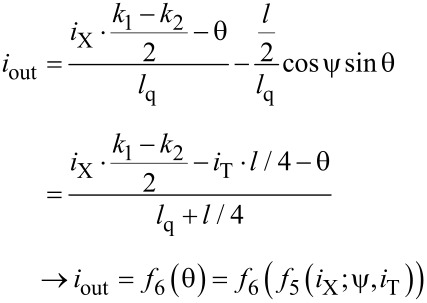


**Figure 5:**
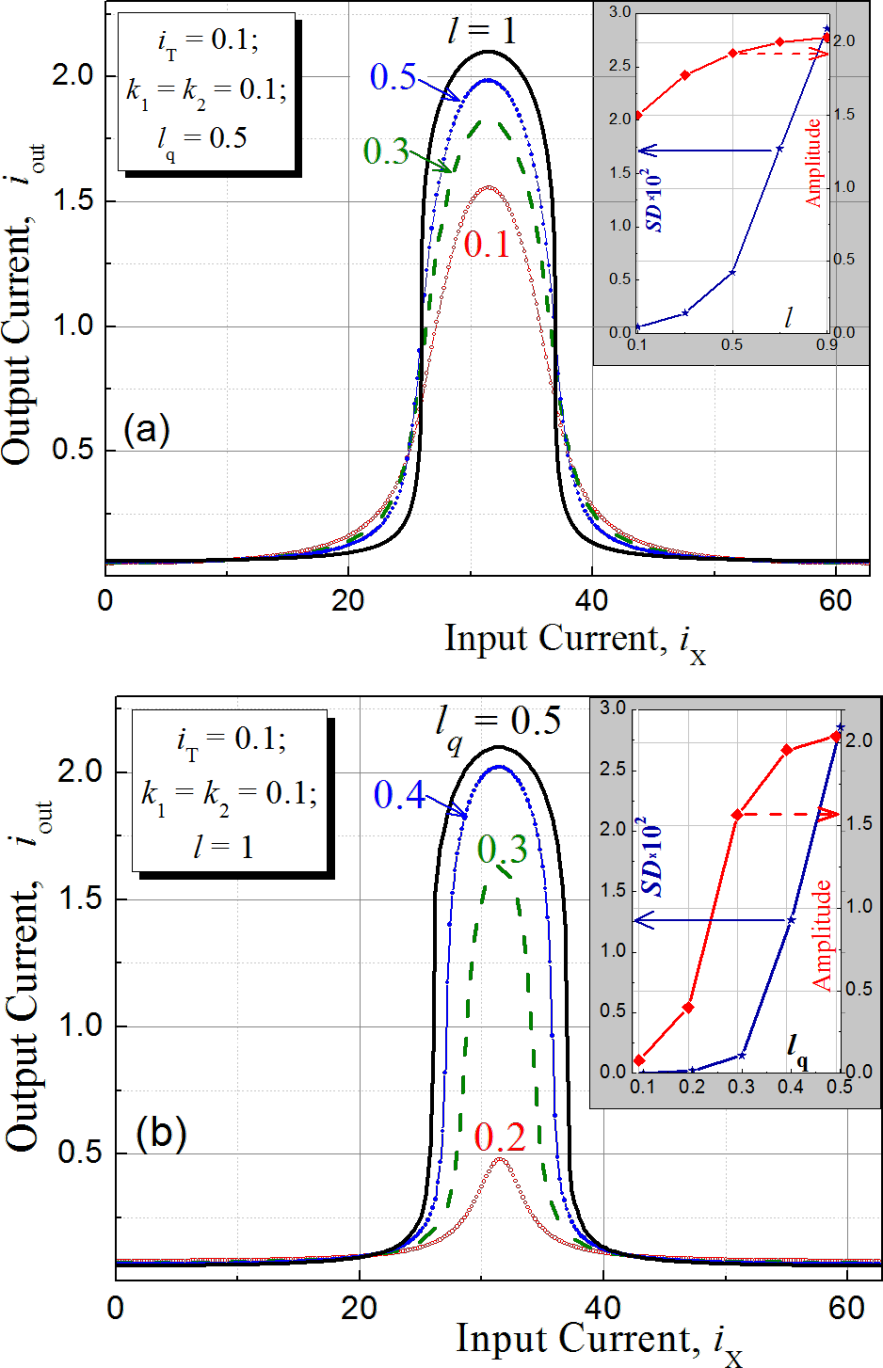
(a), (b) Gauss cell flux-to-current transfer function for different values of the interferometer and shunt inductances *l* and *l*_q_. Insets show the function amplitude and its standard deviation from a Gaussian function.

Here, the term with a linear dependence on the input current cancels in the expression for the output signal (Equation 7). This results in a G-cell flux-to-current transfer function as presented in Figure 5. It is seen that an increase of the normalized inductances *l* and *l*_q_ leads to an increase of the transfer function amplitude and its standard deviation from a Gaussian function.

**Figure 6:**
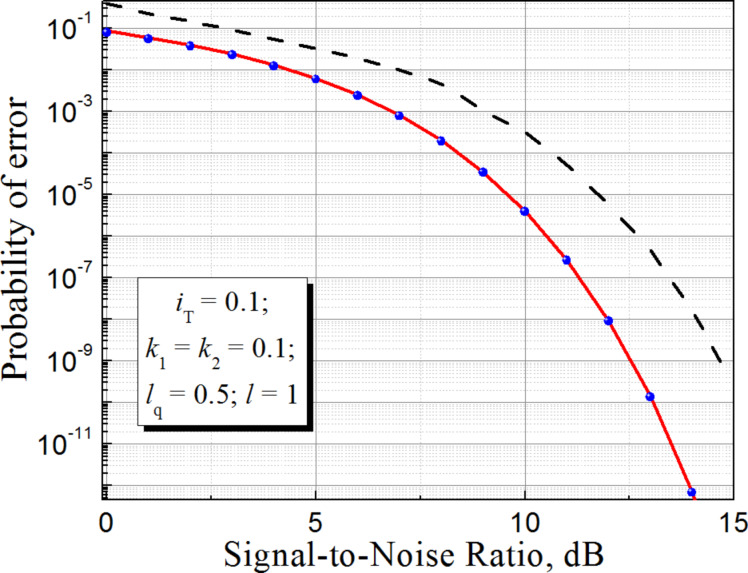
Simulation results for the noise immunity characteristics of a G-cell based RBF ANN with (solid line) and without (dashed line) input data normalization. Dots represent the simulation with an ideal Gaussian activation function.

The simulation results for the noise immunity characteristics of an RBF ANN are shown in Figure 6. Here, the results of the G-cell implementation (with *l* = 1 and *l*_q_ = 0.5 taken in order to get a relatively large output signal) are compared with the ideal case of a true Gaussian activation function of cells in a hidden layer of the probabilistic network.

We should note that the obtained sigmoid-like and Gaussian-like transfer functions are periodic due to the quantization of magnetic flux in superconducting interferometers. This limits the ANN dynamic range. We patch this issue by input data normalization.

In conclusion, we have proposed two superconducting neurons for energy efficient ANNs capable of operation in the adiabatic regime. These ANNs are the most frequently used perceptron and probabilistic RBF network. Consideration of the networks organization and their interface with well-developed adiabatic superconductor logic seems straightforward and will be performed in our upcoming papers.
